# Airway Basal Cells, Protectors of Epithelial Walls in Health and Respiratory Diseases

**DOI:** 10.3389/falgy.2021.787128

**Published:** 2021-11-19

**Authors:** Emma Ruysseveldt, Katleen Martens, Brecht Steelant

**Affiliations:** ^1^Allergy and Clinical Immunology Research Unit, Department of Microbiology, Immunology and Transplantation, KU Leuven, Leuven, Belgium; ^2^Department of Bioscience Engineering, University of Antwerp, Antwerp, Belgium; ^3^Head and Neck Surgery, Department of Otorhinolaryngology, University of Crete School of Medicine, Heraklion, Greece

**Keywords:** airways, basal cells, epigenetics, immune crosstalk, tissue repair, respiratory epithelium

## Abstract

The airway epithelium provides a critical barrier to the outside environment. When its integrity is impaired, epithelial cells and residing immune cells collaborate to exclude pathogens and to heal tissue damage. Healing is achieved through tissue-specific stem cells: the airway basal cells. Positioned near the basal membrane, airway basal cells sense and respond to changes in tissue health by initiating a pro-inflammatory response and tissue repair via complex crosstalks with nearby fibroblasts and specialized immune cells. In addition, basal cells have the capacity to learn from previous encounters with the environment. Inflammation can indeed imprint a certain memory on basal cells by epigenetic changes so that sensitized tissues may respond differently to future assaults and the epithelium becomes better equipped to respond faster and more robustly to barrier defects. This memory can, however, be lost in diseased states. In this review, we discuss airway basal cells in respiratory diseases, the communication network between airway basal cells and tissue-resident and/or recruited immune cells, and how basal cell adaptation to environmental triggers occurs.

## Introduction

**Figure 1:**
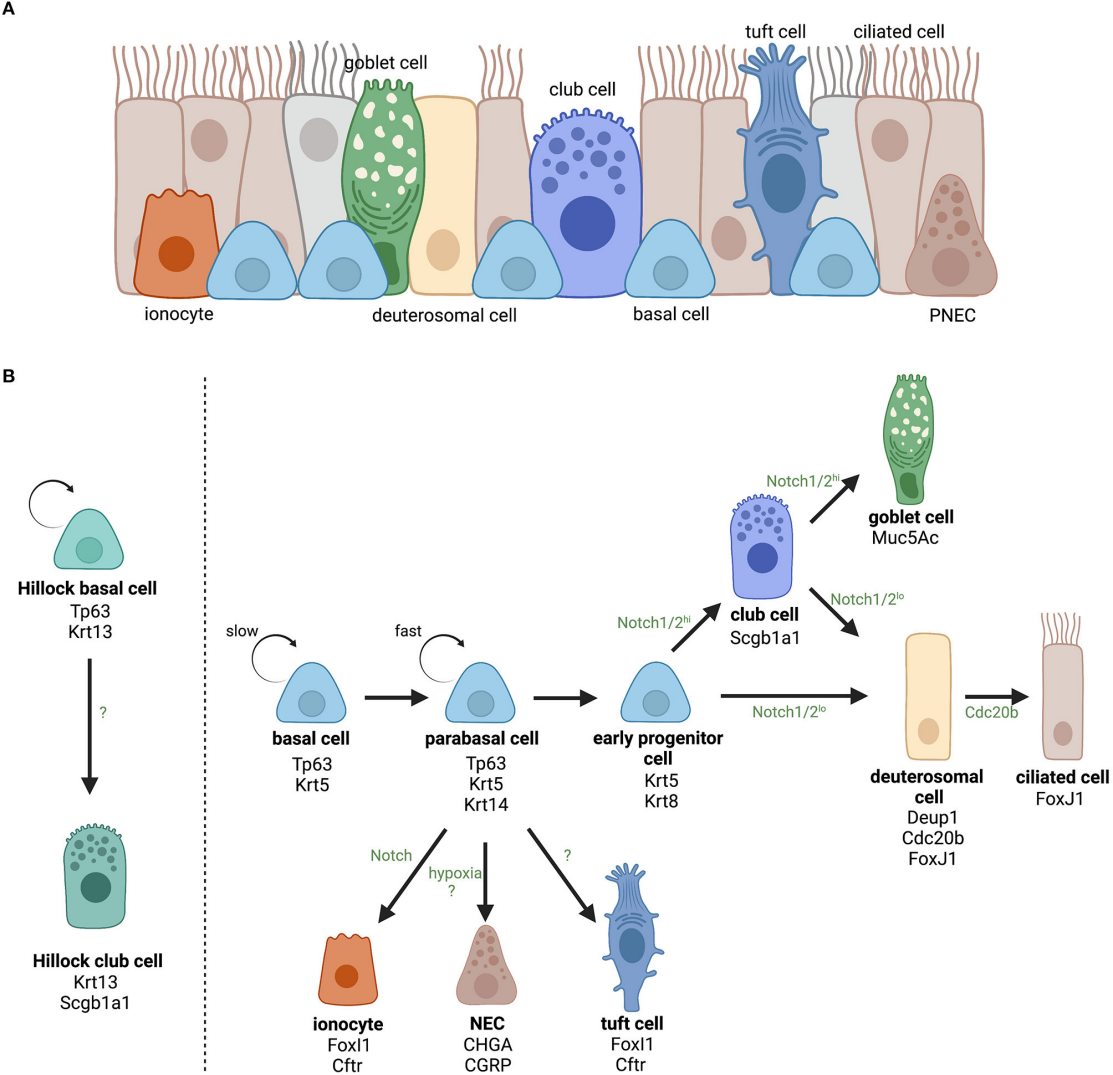
Overview of the identified cell types in the airway epithelium and the current opinion about their lineage hierarchy. **(A)** The airway epithelium is mainly composed of ciliated, goblet and basal cells and more rare cell types including club cells, ionocytes, neuroendocrine cells (NECs), tuft cells, and deuterosomal cells. **(B)** The role of basal cells as progenitor cells of the airway epithelium and the lineage hierarchy of differentiated epithelial cells. For each cell (sub)type, the most important cellular markers are indicated. Upon activation, slowly cycling airway basal cells increase their proliferation rate and become fast cycling parabasal cells. Parabasal cells will continue differentiation and lose expression of basal cell marker Tp63 and gain expression of luminal marker Krt8. Activation of Notch signaling will determine epithelial cell fate toward secretory (club and goblet) or ciliated cells for which the level of Notch2 signaling is decisive (2). Ciliated cell differentiation involves the appearance of a transient state, referred to as deuterosomal cells, characterized by a massive biogenesis of centrioles (i.e., the deuterostome), which is a crucial step in multiciliogenesis (3). On the other hand, airway basal cells can also directly differentiate into ionocytes (FoxI1+ and Ctrf+), NECs (CHGA+ and CGRP+), or tuft cells (Pouf2f2+ and Trpm5+). However, it is still unclear which signaling pathways are involved in those lineages. In addition, in specific squamous epithelial structures termed “Hillocks” Tp63+/Krt13+ basal cells give rise to Scgb1a1+/Krt13+ club cells. Created with BioRender.com.

The respiratory epithelium is the first-line of defense against environmental stimuli such as cigarette smoke, allergens, microbes, and pollutants circulating in the air. This pseudostratified epithelium consists of three main cell types, being ciliated cells, mucus-producing goblet cells, and basal cells (1). However, in recent years, single-cell RNA sequencing (scRNA seq) data has revealed an enormous cellular heterogeneity in the airway epithelium and has provided evidence for novel and/or rarer cell (sub)types in addition to previous histologic data (Figure 1A). Ionocytes, neuroendocrine cells, tuft cells, deuterosomal cells, and club cells (4–8) all have been described, but their function in health and disease is not completely understood. In addition, the distribution and ratio of these different cell types varies along the proximal-distal axis of the airways to meet the local requirements for optimal respiratory functioning, and is often altered in respiratory diseases, including asthma, chronic obstructive pulmonary disease (COPD), and chronic rhinosinusitis with nasal polyps (CRSwNP) (9–11).

To prevent environmental triggers and microbes from entering the submucosa, the epithelium uses an interplay of three defense mechanisms. Firstly, mucus-producing goblets cells secrete mucins that catch environmental triggers entering the airways. In combination with the synchronically organized movements of ciliated cells, mucus-trapped environmental particles are eradicated from the lumen (12). Secondly, the epithelium produces antibacterial (poly)peptides (e.g., lactoferrin, lysozyme) and defensins [e.g., human β-defensin (hBD)-1/2] that directly affect bacterial growth after sensing microbial compounds via specific receptors [e.g., toll-like receptors (TLRs); (13, 14)]. Lastly, neighboring epithelial cells are tightly connected to each other which is essential for the formation of a physical barrier. These connections are mainly formed by two adhesion complexes i.e., tight junctions (TJs) and adherence junctions (AJs). TJs encircle the epithelial cells at the apex and form a proteinaceous seal that regulates paracellular transport of ions, water, and macromolecules (15). They are composed of transmembrane proteins including the families of claudins, occludin, and junctional adhesion molecules that form homotypic/heterotypic interactions to span the intercellular space (16–18). AJs are multiprotein complexes typically containing a classic transmembrane cadherin (e.g., E-cadherin), which is intracellularly connected to the actin cytoskeleton via α- and β-catenin (19). The presence and functionality of these junctional complexes are crucial for maintaining epithelial barrier integrity and their absence or impairment, referred to as barrier dysfunction, is known to cause excessive inflammation of the underlying tissue (9, 20, 21).

To maintain a fully differentiated airway epithelium, epithelial progenitor cells, or airway basal cells continuously monitor airway homeostasis. Basal cells are anchored to the basal lamina via desmosomes and, as a result, are located deeper in the epithelium where they are protected from the external environment. Previous studies, including xenograft models and *in vivo* lineage-tracing, have clearly demonstrated that basal cells are the principal stem cells of the airways with the ability to self-renew post-injury and they are able to differentiate into most other epithelial cell types including columnar ciliated cells, goblet cells, club cells, tuft cells, neuroendocrine cells, and pulmonary ionocytes [Figure 1B; (22–27)]. During epithelial homeostasis, basal cells are relatively quiescent due to the slow turnover of the intact airway epithelium. However, upon injury, basal cells become activated, acquire danger-associated phenotypes (including increased mobility, cytoskeletal rearrangements, deposition of extracellular matrix components) to provide a rapid response and subsequent reconstitution of a fully differentiated epithelium (28). Beside its stem cell-like functions, recent studies have illustrated an interesting role of basal cells in (innate) immunity responses suggesting that their activity and behavior might play a pivotal role in diverse respiratory diseases.

In this review, we will focus on airway basal cell diversity and associated functions, mainly in the upper airways. We will review the cellular interaction between airway basal cells and tissue-resident and recruited immune cells, how basal cells sense environmental stress, and how they can adapt to future insults by learning from previous encounters. Finally, our review concludes with a discussion on the clinical implications of basal cells in health and disease.

## Basal Cell Phenotypes

Basal cells are epithelial progenitor cells and are defined by the expression of transformation-related protein (TP63) and cytokeratins 5 and 14 (KRT5/14) (29, 30). Beside these classical markers, Zhao et al. demonstrated that YES-associated protein 1 (YAP) is essential for the maintenance of airway basal cell identity and that YAP is closely related to TP63 to regulate stem cardinal behavior and to help determine epithelial architecture (31). Depending on their position along the proximal-distal axis, basal cells comprise around 30% of the total airway epithelial cell population in the upper airways and the trachea. The number of basal cells gradually decreases to 6% more distally where they are rather observed in clusters or even as individual cells (32, 33). Although various research groups have studied basal cell numbers along the airways, information about the distribution in the upper respiratory tract, including the nasal cavity, is surprisingly lacking.

In the eighties, Donnelly et al. observed for the first time diversity within the basal cell population, going from quiescent undifferentiated progenitors over intermediate phenotypes, often referred to as parabasal cells, that lost contact with the basal lamina and were shown to contribute to cell renewal (34). Later on, the heterogeneity in differentiation potential was confirmed by *in vivo* lineage-tracing experiments (35) and further uncovered by gene expression analyses (Table 1). Indeed, *Krt14* expression is rather associated with parabasal cells in rat lungs. In the human airways, *KRT14* expression is also more restricted to parabasal cells compared to the more universal basal cell marker KRT5. Using an *in vivo* injury/repair mouse model, Rock et al. demonstrated that after abolishment of nearly all luminal cells, basal cells fuel a well-organized repair process and expand in close proximity to the basal lamina, while maintaining the expression of basal cell-specific markers like Tp63 and Krt5. Interestingly, a new phenotype of parabasal cells was observed after the peak of proliferation that was no longer characterized by the classic basal cell markers. These parabasal cells expressed *Krt8* which is also expressed by columnar epithelial cells [i.e., ciliated and secretory cells; (35, 36)]. The transition from basal to parabasal cell was Notch-dependent and has been confirmed and elaborated on by other research groups (38). Indeed, Notch3 was shown to control the pool of proliferating basal cells and is key to providing a population of parabasal cells that will subsequently differentiate into ciliary or secretory cells by activation of Notch1 and Notch2 (38). How this Notch-mediated basal cell proliferation and differentiation is initiated and suppressed, however, remains uncertain. Other research groups studying the regeneration of the epithelium by basal cells observed even more damage-associated phenotypic changes (28, 37). Basal cells closely located to sites of epithelial injury become activated and acquire mesenchymal cell-associated vimentin, various matrix metalloproteinases (e.g., MMP-3, MMP-9, and MMP-11) necessary for migration toward the wound, squamous cell-associated KRT6, KRT13, and upregulate KRT14 required for the formation of a provisional barrier (28, 37). Recently, a novel epithelial structure containing many Krt13^+^ cells led to the identification of a new cell type, namely Scgb1a1^+^ Krt13^+^ club cells (8). These structures are characterized by high turnover rates and were termed “Hillocks.” In these “Hillocks,” a distinct subgroup of basal progenitor cells expressing both *Tp63* and *Krt13*, referred to as Hillock basal cells, were identified that specifically give rise to these Hillock club cells (8). The function of Hillocks and Hillock basal cells in health, however, is not discovered thus far.

**Table 1:** Overview of currently identified airway basal cell subtypes.

**Specific markers**	**Functional annotation**	**References**
TP63+ KRT5+	Quiescent progenitor cell	(29, 34)
TP63^+^ KRT5^+^ KRT14^+^	Parabasal cell, proliferative columnar progenitor cell	(33, 34)
TP63^−^ KRT8^+^	Parabasal cell, proliferative columnar progenitor cell	(35, 36)
TP63^+^ KRT5^−^ KRT14^+^	Hillock basal cell, progenitor Hillock club cell	(8)
TP63^−^ KRT6^+^ KRT13^+^ KRT14^+^ vimentin^+^	Motile basal cells, formation provisional barrier	(37)

It is clear that (para)basal cells are not a uniform cell population. More recently, single-cell RNA sequencing experiments nicely revealed the basal cell trajectory toward terminally differentiated cells using diffusion pseudotime mapping, illustrating the diversity of basal cell intermediate phenotypes (5). As their data also suggested impairment of differentiation potential of basal cells in polyp tissue of CRS patients compared to non-polyp tissue. Ordovas-Montanes et al. additionally performed assays for transposase-accessible chromatin (ATAC) sequencing to identify intrinsic epigenetic changes. This revealed that epigenetic alterations lie at the basis of the pathologic phenotype of basal cells in CRSwNP and provided the first evidence for immune memory in airway epithelial progenitor cells in analogy to what was previously shown in the skin (39). Today, pathologic phenotypes of basal cells such as basal cell hyperplasia and metaplasia have been identified in multiple inflammatory conditions of the airways, including asthma, COPD and cystic fibrosis, and are believed to play a critical role in their pathogenesis (40–44).

## Sensing Tissue Damage and Orchestrating Epithelial Repair After Injury

As stated previously, the airway epithelium has the important function to provide a physical barrier to prevent the infiltration of potential threats. However, the continuous passage of chemical compounds, microbes, and airborne particles can potentially damage the epithelium, making the underlying tissue vulnerable for infections. In order to prevent more harm, tissue damage need to be resolved as soon as possible. Airway epithelial injuries can be sensed by the remaining neighboring epithelial cells, resulting in the production of alarmins, damage-associated phenotypic changes of the basal cells, and the initiation of tissue repair mechanisms. As a result, immune cells are triggered to generate an appropriate response and facilitate resolution and tissue homeostasis. In this part, we will focus on the mechanisms behind tissue repair after injury.

During normal tissue homeostasis, the airway epithelium has a rather slow turnover rate (30–50 days) in comparison with, for example, the intestinal epithelium (3–5 days). To maintain this quiescent state, airway epithelial cells express anti-inflammatory compounds, such as SCGB1A1 and IL-37, that attenuate the expression of pro-inflammatory cytokines and have been shown to lower allergic airway inflammation (45, 46). When the epithelium is injured, it reacts vigorously to reestablish the breached barrier with resident cells as the source of the new cell population. Indeed, upon damage, the neighboring epithelial cells will undergo phenotypic changes, due to loss of contact inhibition from neighboring cells and an increasing gradient of factors (e.g., cytokines and growth factors) from the wound site, which enable them to migrate over and seal the wound (47). Gene expression analysis of those spreading cells in human showed that these cells were positive for vimentin and KRT14, illustrating the important role of basal cells in the initial phase after injury (48, 49). Interestingly, secretory epithelial cells (i.e., goblet and club cells) have the capacity to dedifferentiate in absence of basal cells and become functional TP63^+^ and KRT5^+^ airway progenitor cells (49–51). In the small airways (i.e., bronchioles), dedifferentiation of these cells is the main source of tissue repair, as basal cell numbers are particularly low in human or even absent in murine small airways (50). In the alveoli, several cell populations are identified as alveolar stem cells/progenitors involved in regeneration, including bronchioalveolar stem cells (BASCs) (52–54), alveolar type II cells (AT2s) (55, 56), and basal cell-like TP63^+^ lineage-negative epithelial progenitor cells (LNEPs) (57–59). The motile phenotype of those migratory cells is established by rearrangements of the actin cytoskeleton and related integrins (e.g., α5β3), resulting in polarization and the formation of lamellipodia and filopodia (47). In combination with other environmental changes (e.g., loss of cell contacts upon injury), transforming growth factor (TGF)-β, produced by damaged epithelial cells, and increased β-catenin signaling are the driving force behind this injury-initiated epithelial to mesenchymal transition (EMT) (60, 61). However, other studies propose an inhibitory role for TGF-β on tissue repair as it is a major promoting factor of fibrosis (62–64).

The migration of progenitor cells is facilitated by the degradation and subsequent modification of the extracellular matrix (ECM), a process referred to as ECM remodeling. The degradation is primarily performed by matrix metalloproteinases (i.e., MMP-3, MMP-9, and MMP-11), that are mainly released by the migratory basal cells (28, 37). In addition, those migratory basal cells also deposit new ECM compounds that ease their migration and provide a temporary provisional barrier that covers the wound in attendance of full resolution, a mechanism that is again proposed to be initiated by TGF-β (48, 65). While colonizing the denuded epithelium, the migratory progenitor cells start to proliferate to completely cover the damaged site. Once at place, they differentiate to generate a functional pseudostratified epithelium which can continue for days to even weeks. Multiple factors have been identified to stimulate proliferation, but the activation of EGFR on the repair cells is believed to play the leading role (66). EGFR can be stimulated by AREG, produced by basal repair cells (autocrine regulation) as well as ILC2s and Tregs, but also in a ligand-independent way by oxidative stress generated by cigarette smoke or neutrophils (67–69), illustrating the extensive capacity of EGFR to regulate basal cell proliferation. In addition, WNT signaling in airway epithelial progenitors also controls the regeneration after injury (70). In the trachea of adult mice, canonical Wnt signaling is activated within basal cells after damage, stimulates proliferation, and directs their differentiation into ciliated or secretory cells (71, 72). Similar observations were made for other epithelial progenitor cells (i.e., club cells, BASCs, and AT2) in other regions of the airways, emphasizing a universal role for WNT signaling in tissue repair along the airways (70, 73, 74). Besides, basal cell differentiation and proliferation is also shaped by activation of Notch1 and Notch2 in response to Notch3 stimulation, as stated previously in this review (38). In addition, various molecules have been identified to be able to modulate differentiation of airway basal cells, including insulin growth factor (IGF)-1 and interleukin (IL)-13 (75, 76).

It is clear that basal cells are essential players in tissue repair after injury and that their activity is tightly regulated by a wide variety of control mechanisms and signaling pathways. Although this complex phenomenon has been studied for decades now, it is still not yet completely understood.

## The Basal Cell—Immune Cell Crosstalk

The last decades, our understanding of how the airway epithelium can induce and regulate immune responses upon encountering environmental triggers grows enormously (77–80). The question, however, remains how the airways remain protected from infection in case of epithelial cell damage and when basal cells become exposed to potential threats. An answer was provided by Amatngalim et al., who demonstrated that basal cells, beside their function as stem cells/progenitors in epithelial regeneration, also serve as a unique source of host defense (81). Stimulation of basal cells with *Haemophilus influenzae*, a common respiratory pathogen, resulted in the upregulation of the antimicrobial protein RNase7, pro-inflammatory cytokines IL-6, IL-8, and other innate immune mediators. In addition, it was shown that *Pseudomonas aeruginosa* binds α5β1 integrin (82), rhinoviruses attach to intercellular adhesion molecule (ICAM-)1 (83), and respiratory syncytial virus (RSV) infects the cells via ICAM-1 and/or EGFR recognition (84, 85), illustrating that airway basal cells are a target for multiple respiratory pathogens. These infections drive basal cell fate toward mucus-producing secretory cells to promote a faster eradication of pathogens from the airways. With the help of emerging novel technologies, new insights have been gained regarding the specific role of basal cells in immunity and intercellular communication. In this section, we will focus on the crosstalk between basal cells and immune cells, including macrophages, innate lymphoid cells (ILCs), and regulatory T cells.

### Macrophages

Macrophages are the primary initiators of innate immunity in the lung and possess a large arsenal of immune receptors (86, 87). Traditionally, two distinct subsets of macrophages are defined, M1 and M2 macrophages (88). M1 macrophages have a pro-inflammatory character and are involved in pathogen removal. M2 macrophages are more associated with parasite defense and tissue repair (89). Novel studies, however, demonstrated that a wide spectrum of various macrophage phenotypes exists (90).

**Figure 2:**
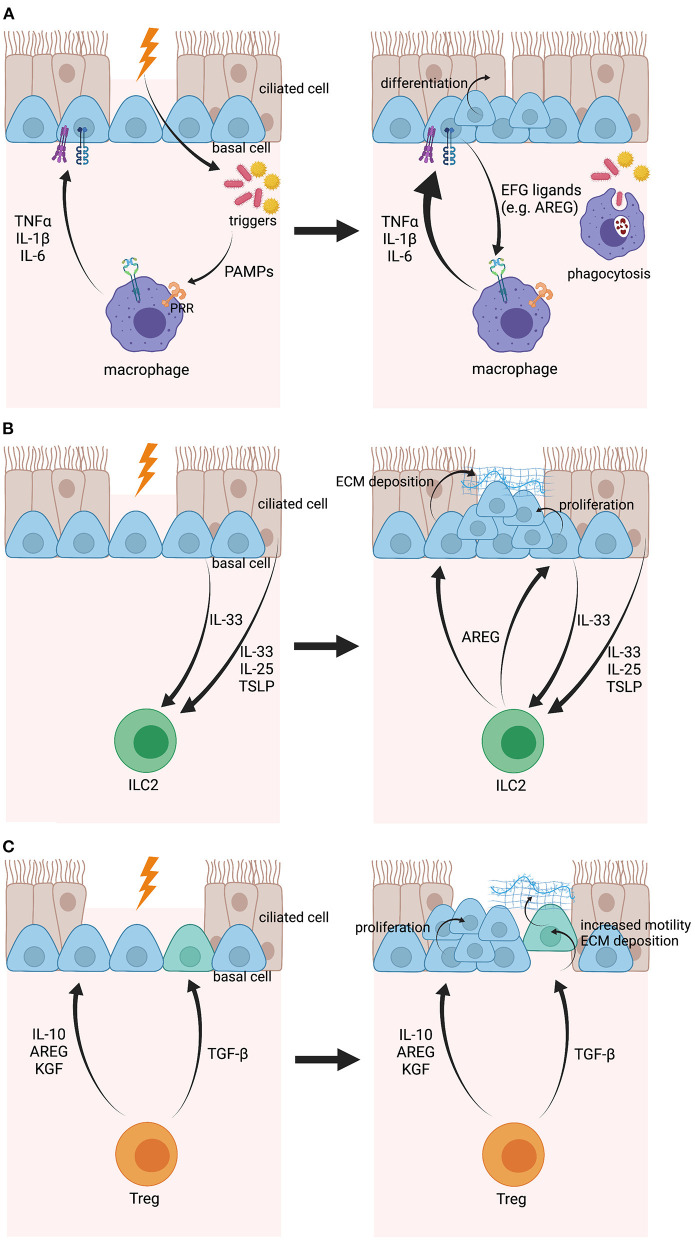
Illustration of the crosstalk between airway basal cells and macrophages, ILC2s and Tregs, respectively, upon epithelial damage. **(A)** When the epithelial barrier is damaged, pathogens, or other environmental triggers can enter the subepithelial space and will be sensed by macrophages via PRRs. Macrophages become activated and express pro-inflammatory mediators, including IL-1β, TNFα, and IL-6, that will be sensed by airway basal cells via their respective receptors and will encourage basal cell differentiation. In their turn, airway basal cells will produce EGF ligands that boost macrophage activation even further. **(B)** Airway basal cells and other epithelial cells can sense epithelial damage when in close proximity and will express IL-33, IL-25, and TSLP as a response. These mediators will then activate ILC2s and stimulate their expression of AREG. AREG will be sensed by airway basal cells and will lead to ECM deposition and proliferation of basal cells. **(C)** Tregs are activated after epithelial damage and produce TGF-β, IL-10, AREG, and KGF. While TGF-β will induce EMT in basal cells, leading to increased motility and ECM deposition to move over the wound and provide a provisional barrier, IL-10, AREG, and KGF will stimulate basal cell expansion before full restoration of the epithelium. Created with BioRender.com.

In the context of breached barriers, macrophages, and airway epithelial cells work closely together to promptly restore epithelial barrier integrity (Figure 2A). It is known that columnar epithelial cells express a wide variety of pathogen-recognition receptors (PRRs) that can sense the presence of microbial triggers in the lumen and subsequently promote the production of cytokines (e.g., IL-6, IL-8), defensins (e.g., h-BD1), and alarmins [e.g., TSLP, IL-33, and IL-25; (91)]. In case of a breached barrier, the trigger can undermine the epithelial barrier and reach the submucosa, where it will stimulate and activate macrophages by binding their PRRs (92). As a response, macrophages upregulate their expression of cytokines, including tumor necrosis factor α (TNFα), IL-1β, and IL-6, which are then recognized by their receptors on epithelial cells to further induce their production of cytokines, defensins, and alarmins and to upregulate epithelial TLR expression resulting in increased reactivity of the epithelium to pathogens (93). In most of these studies, the epithelium was seen as a whole entity, without a clear understanding how specific subgroups of epithelial cells react. Transcriptome analysis of human airway basal cells has demonstrated cell-specific insights in how basal cells communicate with macrophages. More specifically, basal cells express a surprisingly broad spectrum of epithelial growth factor (EGF) family ligands, including amphiregulin (AREG), epiregulin, and neuregulin, which can stimulate the EGF receptors (EGFR) on macrophages, leading to macrophage activation and cytokine production via induction of the nuclear factor kappa-light-chain-enhancer of activated B cells (NFκB) and mitogen-activated protein kinase (MAPK)1/3 pathways (94, 95). In addition, members of the TNF receptor (TNFR) and IL-1 receptor (IL-1R) protein families have been shown to be enriched in basal cells compared to the differentiated epithelium, indicating that basal cells are important in sensing macrophage-produced immune mediators (95). In that perspective, IL-6, a major M1 macrophage cytokine, promotes the regeneration of ciliated cells from basal cell progenitors in the airways via signal transducer and activator of transcription (STAT)3 activation (96). Further evidence for a basal cell-macrophage axis was delivered by Engler et al., who discovered a population of C-C chemokine receptor type 2 (CCR2)-expressing monocytes, a progenitor of monocyte-derived macrophages, that live in very close proximity to airway basal cells and appeared to be necessary for efficient epithelial repair after chemical injury using polidocanol (97). It remains unknown whether macrophages play a role in epithelial homeostasis during steady-state, though, it is believed that alveolar macrophages are kept quiescent via cell-to-cell contacts with epithelial cells (e.g., gap junctions) and communicate via extracellular vesicles (98). Taken together, the crosstalk between basal cells and macrophages is essential to accelerate tissue repair. However, how these two cell types activate/control each other's function in health is not completely understood.

### Innate Lymphoid Cells

Innate lymphoid cells (ILCs) are relatively recently discovered non-B non-T lymphocytes that originate from a common lymphoid progenitor but lack specific antigen-receptors. They are described as the innate counterpart of adaptive T cells, although, in contrast to T cells, ILCs are largely tissue resident cells, deeply integrated in the residential tissues, and are only rarely observed in primary or secondary lymphoid organs (99). ILCs are especially abundant at barrier surfaces of the lung, skin and gut, and thus support their capacity to quickly react in case of tissue disturbances (100–102). ILCs are classified in three main groups: type 1, type 2, and type 3 ILCs, which has been nicely reviewed previously (99, 103, 104).

Considering their proximity with tissue barriers, ILCs play an important role in controlling tissue homeostasis, immunity against infections and induction of tissue repair (105). When focusing on tissue repair, the main role is reserved for ILC2s, which are directly activated by epithelial-derived alarmins, such as IL-25, IL-33, and TSLP [Figure 2B; (106–109)]. Indeed, Monticelli et al. demonstrated that ILC2s promote lung tissue homeostasis after influenza infection in response to IL-33 stimulation and that depletion of ILC2 strongly impairs tissue regeneration (110). Other studies showed that a subset of airway basal cells function as an important source of IL-33 during inflammation (111). Using a post-viral mouse model for COPD, Byers et al. illustrated that Krt5^+^, Tp63^+^, and Krt14^+^ basal cells produce IL-33 after Sendai virus infection. Similar observations were made in whole lung explants from COPD patients undergoing lung transplantation (111). Subsequently, IL-33 stimulates ILC2s to produce AREG, which will induce basal cell proliferation, an essential early step in the process of tissue regeneration upon damage (112). However, overactivation of ILC2 and AREG production might eventually lead to basal cell hyperplasia, which has been allocated as one of the mechanisms mediating the pathogenesis behind smoking-induced lesions (112). In addition, AREG has been associated with fibrosis in the lungs, liver, and skin (108, 112, 113), due to excessive ECM deposition by epithelial cells. Although it was not specified which type of epithelial cell(s) is/are responsible, it is known that basal cells increase ECM production upon injury to generate a provisional barrier in an early response (37, 114, 115).

Taking together, it is clear that airway basal cells establish a powerful crosstalk with ILC2s that requires strict regulations as a disbalance can result in pathologic remodeling of the resident tissue. At this point, there is no evidence for a direct interaction between airway basal cells and other ILC subtypes.

### Regulatory T Cells

Regulatory T cells (Tregs) were originally identified as a small subset of CD4^+^ immune cells that play a central role in tolerance and suppression of autoimmunity (116, 117). They are subdivided in two main groups: (1) natural Tregs (nTregs), that are generated in the thymus during conventional T cell development, and (2) induced Tregs (iTregs), that arise in the peripheral circulation after induction of other T cell subtypes with TGF-β (118, 119). Besides CD4, Tregs express surface marker CD25 and are characterized by transcription factor forkhead box p3 (FoxP3), which is needed for the release of anti-inflammatory cytokines such as IL-10, IL-35, and TGF-β to control immune responses (120–123). Tregs have the capacity to suppress the proliferation and/or activation of a wide variety of immune cells (e.g., CD4^+^ T cells, CD8^+^ T cells, B cells, and NK cells) via multiple mechanisms, including cell-to-cell contact-dependent suppression, cytokine production, and perforin/granzyme-mediated cytotoxicity (124).

There is increasing evidence that Treg function extends beyond being a sole damper of inflammatory responses. In multiple tissues (i.e., skin, intestine, lung, and muscle), it has been demonstrated that Tregs are important mediators of tissue or wound repair [Figure 2C; (125, 126)]. In particular, a subtype of Tregs expressing high levels of AREG has been identified shortly after injury and depletion of this population in a mouse model of acute lung injury results in prolonged and impaired tissue regeneration (127, 128). Mock et al. discovered that epithelial proliferation coincided with an increase of FoxP3^+^ Tregs during the course of resolution in an experimental model of acute lung disease (129). They later identified the soluble molecule produced by Tregs as keratinocyte growth factor (KGF) as it was responsible for enhanced primary epithelial cell proliferation *ex vivo* (130). In addition, Ali et al. demonstrated in the skin that Tregs also facilitate epithelial stem cell proliferation and differentiation by expressing jagged1 (Jag1), which is then sensed by Notch receptors on hair follicle stem cells (HFSC) (131). Furthermore, anti-inflammatory IL-10 is also put forward as a potential mediator for stem cell function. Using intestinal organoids, it has been shown that both cocultures with Tregs and treatment with its main cytokine IL-10 result in intestinal stem cell expansion, while cocultures with other Th cell subgroups or cytokine treatments caused a decrease of intestinal stem cell numbers (132). Lastly, TGF-β induces phenotypic changes in basal cells upon injury, leading to more motile progenitors, and deposition of ECM compounds in order to generate a provisional barrier that covers the wound (48, 65).

Compared to other tissues, studies on the interaction between Tregs and airway basal cells are lagging behind. Future research is necessary to further clarify the crosstalk and its role in regulating epithelial homeostasis.

## Inflammatory Memory of Airway Basal Cells

The capacity of the airway epithelium to respond to a broad spectrum of pro-inflammatory triggers by initiating an appropriate defense reaction has already been studied for years (79, 80). Airway epithelial cells can adapt their transcription profile when sensing cytokines and it was, for example, shown for IL-4 and IFN-γ that this occurs in both a synergistic and antagonistic way (133). Recently, a new dimension regarding this protective function has been uncovered. Indeed, there is growing evidence that the airway epithelium can generate memory after contact with a specific trigger (e.g., an allergen, pathogen, or chemical). ScRNA sequencing experiments in combination with ATAC sequencing showed that IL-4 and IL-13 responsive genes were upregulated in basal cells from patients with CRSwNP compared to non-polyp patients and that those basal cells maintain an undifferentiated state due to an upregulation of transcription factors such as KLF5 and ATF3 (5). Interestingly, ATF3 upregulation is also observed during viral infection and house-dust mite allergy (134). Moreover, stimulation of isolated basal cells from both patient populations showed that basal cells from polyp tissue upregulated a 10 times higher number of genes compared to non-polyp tissue (i.e., ethmoid sinus tissue of patients spanning the CRS spectrum) and that levels of Wnt pathway activator CTNNB1 at baseline in polyp tissue could only be reached after IL-4/IL-13 stimulation in non-polyp tissue, indicating intrinsic changes on the epigenetic level (5). These observations are in line with previous studies in skin and intestine, where post-inflamed mice showed faster healing after wounding compared to naïve mice and organoids from intestinal epithelial stem cells of mice on a high-fat diet grew abnormally, respectively (39, 135). Further indications for inflammatory memory in airway epithelial cells include the observation that barrier and junctional defects as well as ciliated cell hypoplasia persist in air-liquid interface (ALI) airway epithelial cell cultures from smoker and COPD patients (136). In addition, Martin et al. demonstrated *in vitro* that human bronchial epithelial cells (i.e., BEAS-2B cell line) remember infection as they can induce either a trained or tolerant response, dependent on the combination of primary and secondary trigger, meaning that their secondary response was modulated after the initial trigger (137). Moreover, they showed that inhibitors of histone acetyltransferase (EGCG) and histone methyltransferase (BIX) can abolish the IL-8 trained immune response without affecting IL-8 expression. This finding suggests that immune memory of epithelial cells is likely regulated via histone modifications and chromatin accessibility, an observation that was also supported by the ATAC sequencing data form Ordovas-Montanes et al. (5) and Martin et al. (137). The ability of non-immune cells to install an inflammatory memory is a rather new concept. It was first believed that this concept was restricted to adaptive (memory T and B cells) and innate (macrophages, ILCs, natural killer cells) immune cells (138). However, emerging groundbreaking data proposing the memory potential of tissue stem cells, fibroblasts and microglial cells are changing our view on immunity (139).

## Discussion

It is clear that the function of airway basal cells extends much further than simply being the epithelial progenitor cells and that the phenotypic diversity of this cell population facilitates their involvement in various processes. In normal tissue homeostasis, the airway basal cells remain rather quiescent, while in case of tissue disturbances they are able to sense damage and/or intruders, undergo phenotypic changes, and respond properly. In case of epithelial injury, we discussed that basal cells undergo EMT in response to TGF-β, characterized by the expression of vimentin and MMPs, flattening of the cell and increased mobility, and reactivate cell cycling and proliferation. These features are crucial for adequate tissue repair, but requires very strict regulation, as this might lead to pathogenicity when disturbed. This is observed in multiple airway diseases, including COPD and asthma. In COPD, the airway epithelium displays features of dedifferentiation toward mesenchymal cells, which correlate with peribronchial fibrosis and airflow limitation, and this has been partly allocated to TGF-β-driven epithelial reprogramming (140). Another feature in COPD is basal cell and goblet cell hyperplasia. As we discussed, Notch signaling is an important controller of basal cell differentiation and certain key genes of the Notch pathway are decreased in smokers and COPD patients compared to healthy individuals (141). In case of asthma, accumulation of TGF-β in the bronchoalveolar fluid and increased sensitivity of the asthmatic basal cells to TGF-β suggest an unmistakable role for dysregulated EMT in tissue remodeling and impaired barrier function (142). With regard to the upper airways, single-cell sequencing data illustrated that basal cells in polyp tissue of CRSwNP patients are stuck in an undifferentiated state and intermediate populations of parabasal cells are decreased compared to non-polyp tissue, leading to impaired differentiation potential of the epithelium and basal cell hyperplasia (5).

While it is still a field in its infancy, cellular memory of previous experiences that influence future responses has been reported for several diverse stem cell populations (5, 39, 135). This type of memory is a well-known feature of the adaptive responses seen in immune cells. Observing trained inflammatory responses by non-immune cells is a new emerging field in immunology. Although the installation of such cellular memory by basal cells can accelerate the response to a second wound, the danger exists that persistent exposure to inflammatory cells and signals can imprint a negative epigenetic memory that can delay tissue regeneration and promote disease chronicity. For example, it is suggested that epithelial barrier dysfunction, a feature observed in several respiratory conditions including CRS, allergy and asthma, is imprinted in the basal progenitor cells as a result of continuous exposure to type 2 inflammatory agents, and in that way conserved over time (20, 143–146). The idea that disease chronicity is imprinted in basal cells was also suggested in other epithelial tissues, including skin and intestine (147, 148). As basal cells are the main progenitors to restore the epithelium, the question raises whether these cells can be used in regenerative stem cell therapy. Although this might sound promising, this field requires extensive fundamental research focusing on basal cell phenotypes in health and disease prior to studying the therapeutic potential. Some first encouraging results were delivered by Steelant et al., who showed an equal proliferative and differentiation capacity of basal cells from cystic fibrosis patients compared to healthy controls, which supports the feasibility of autologous cell therapy for cystic fibrosis lung disease (149).

On the other hand, the existence of a cellular memory raises the prospect of devising therapeutic agents that might mimic the molecular underpinnings of positive regenerative memories or erase the bad memories that occur in chronic conditions. In that light, the use and effects of nanoparticle technology on the epigenome has been studied over the last years (143–146). Although evidence suggests that epigenetic modifications can be induced by nanoparticles, the mechanisms behind these processes remain largely unclear, and raises concerns regarding nano- and epigenetic toxicity.

## Author Contributions

ER drafted the manuscript. KM and BS critically revised the manuscript. All authors contributed to the article and approved the submitted version.

## Funding

KM was supported by a Postdoctoral Fellowship of the Fund of Scientific Research (FWO, 12Z0622N), Flanders, Belgium. BS was supported by a Postdoctoral Fellowship of the Fund of Scientific Research (FWO,12U6721N), Flanders, Belgium.

## Conflict of Interest

The authors declare that the research was conducted in the absence of any commercial or financial relationships that could be construed as a potential conflict of interest.

## Publisher's Note

All claims expressed in this article are solely those of the authors and do not necessarily represent those of their affiliated organizations, or those of the publisher, the editors and the reviewers. Any product that may be evaluated in this article, or claim that may be made by its manufacturer, is not guaranteed or endorsed by the publisher.
